# Supplementary figures and images for: Proteome-wide identification of HSP70/HSC70 chaperone clients in human cells
Source: PLoS Biol. 2020 Jul 20;18(7):e3000606. doi: 10.1371/journal.pbio.3000606 (PMC7392334; doi:10.1371/journal.pbio.3000606)

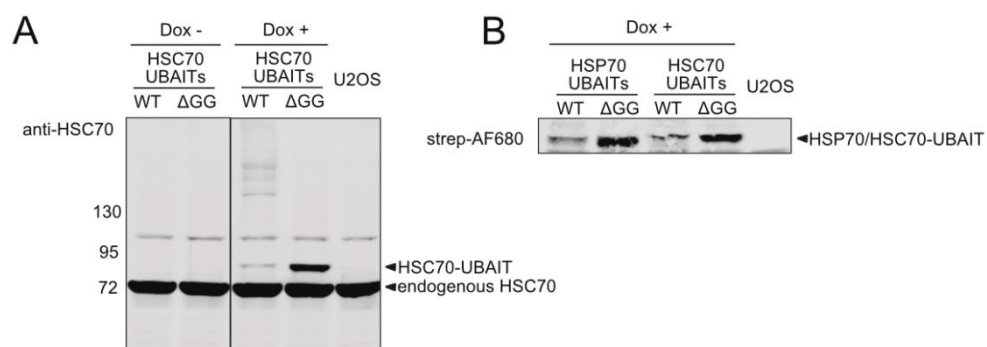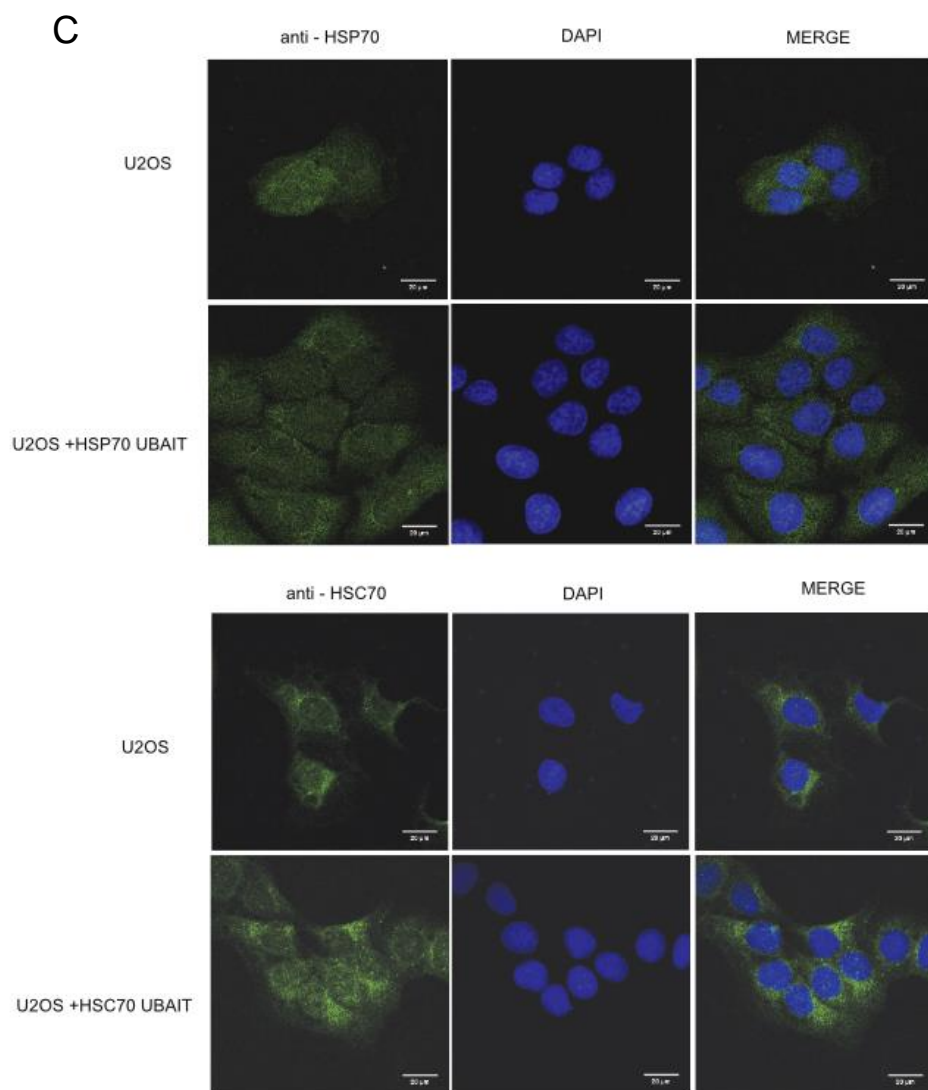

Supplement: S1 Fig — (A) Western blot of HSC70 UBAITs expressed in human U2OS cells treated with doxycycline (Dox) (1 ug/mL) for 3 days or untreated, using anti-HSC70 (Santa Cruz sc-7298). (B) Western blot of HSP70/HSC70 UBAITs expressed in human U2OS cells treated with Dox (1 ug/mL) for 3 days or untreated, using streptavidin-AlexaFluor680 (Life Technologies). (C) U2OS cells and U2OS cells expressing the ubiquitin-tagged UBAIT constructs (with doxycycline) were analyzed by immunofluorescence using antibodies directed against HSP70 (Enzo ADI-SPA-810) or HSC70 (Santa Cruz sc-7298) and imaged by confocal microscopy, with DAPI as the counterstain, as indicated. HSC, heat shock cognate; HSP, heat shock protein; UBAIT, ubiquitin-activated interaction trap. (PDF) [file pbio.3000606.s001.pdf]

*Δssa1-4*

+ vector

+ SSA1  
UBAIT ΔGG

+ wild-type  
SSA1

+ SSA1  
UBAIT

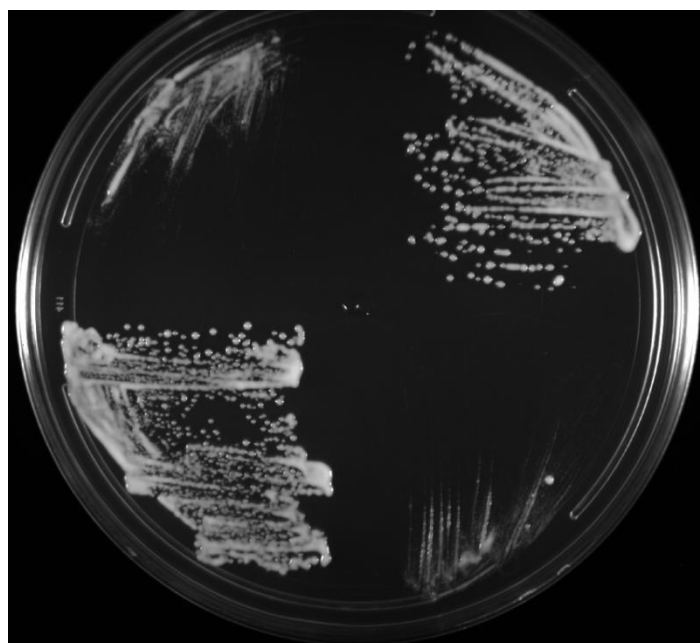

Supplement: S2 Fig — An S. cerevisiae strain deficient in SSA1, SSA2, SSA3, and SSA4 was complemented by vector only, wild-type SSA1, SSA1 UBAIT (C-terminal ubiquitin fusion), or SSA1 UBAIT ΔGG (C-terminal ubiquitin fusion lacking GG at the C terminus), as indicated. Strains were streaked onto 5-FOA media, which selects for loss of the URA3 (wt SSA1) plasmid, maintaining viability of the ssa1-4 strain. See also S6 Data. SSA1, stress-seventy subfamily A 1; UBAIT, ubiquitin-activated interaction trap; wt, wild type; 5-FOA, 5-Fluoroorotic acid. (PDF) [file pbio.3000606.s002.pdf]

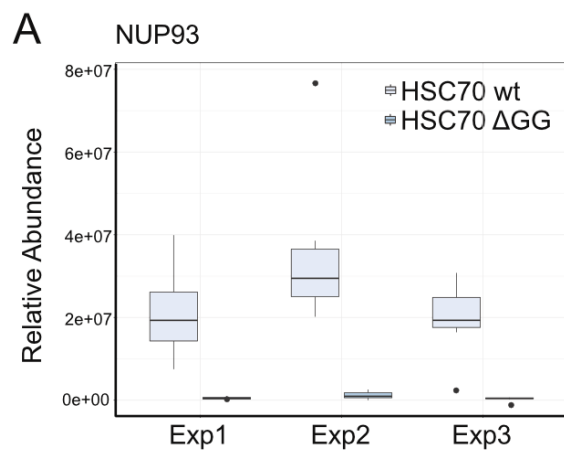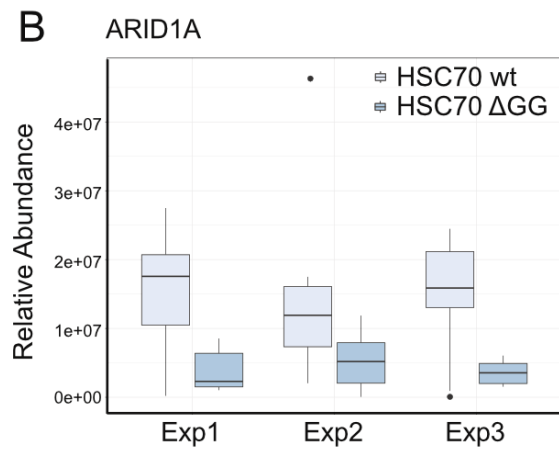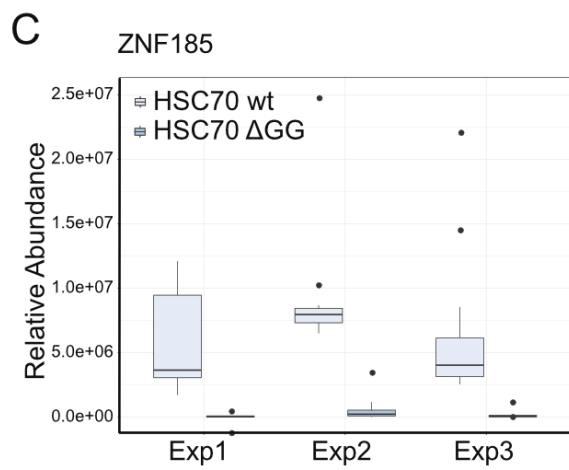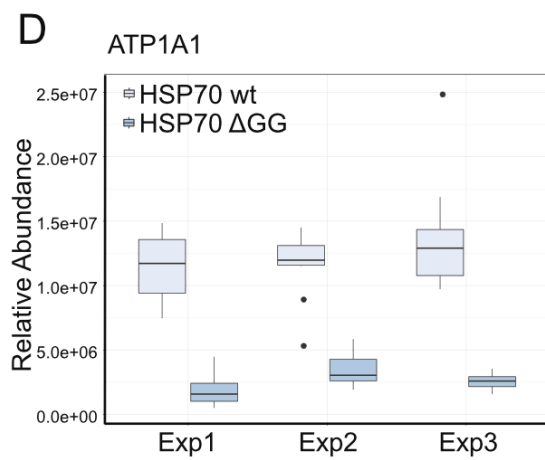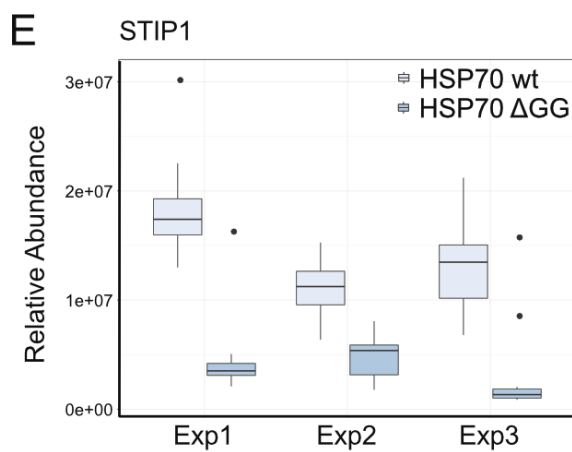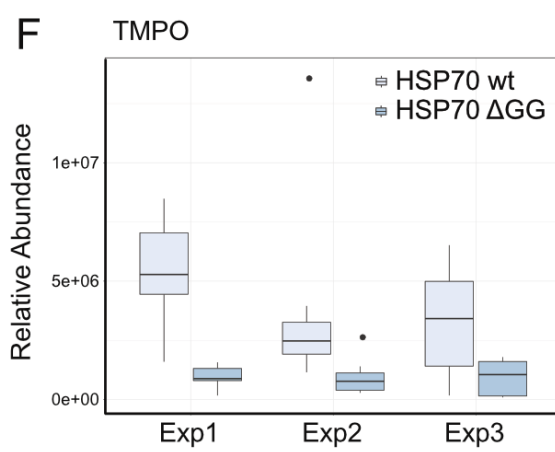

Supplement: S3 Fig — (A, B, C) Levels of binding of specific targets to HSC70 UBAIT wild-type or ΔGG isolations (normalized by level of HSC70 expression), with 12 replicates shown per sample. (D, E, F) Levels of binding of specific targets to HSP70 UBAIT wild-type or ΔGG isolations (normalized by level of HSP70 expression). All examples shown yield enrichment values that exceed the 95% confidence interval and are retained at FDR 0.05 using Benjamini-Hochberg (see Materials and methods for details). FDR, false discovery rate; HSC, heat shock cognate; HSP, heat shock protein; UBAIT, ubiquitin-activated interaction trap. (PDF) [file pbio.3000606.s003.pdf]

## HSC70

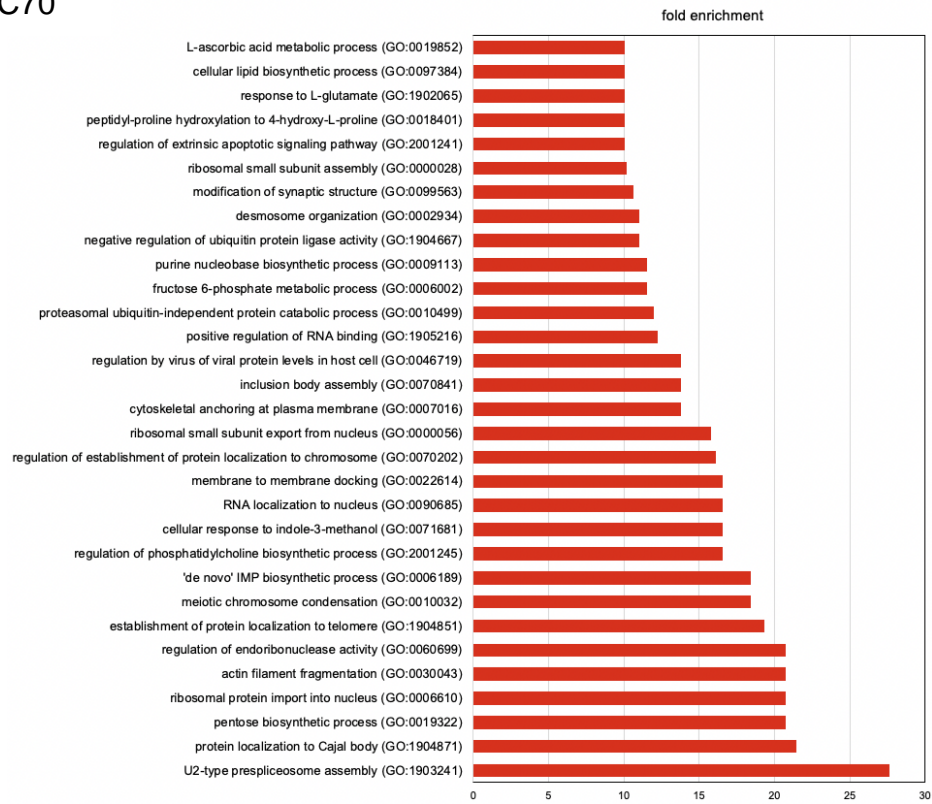

## HSP70

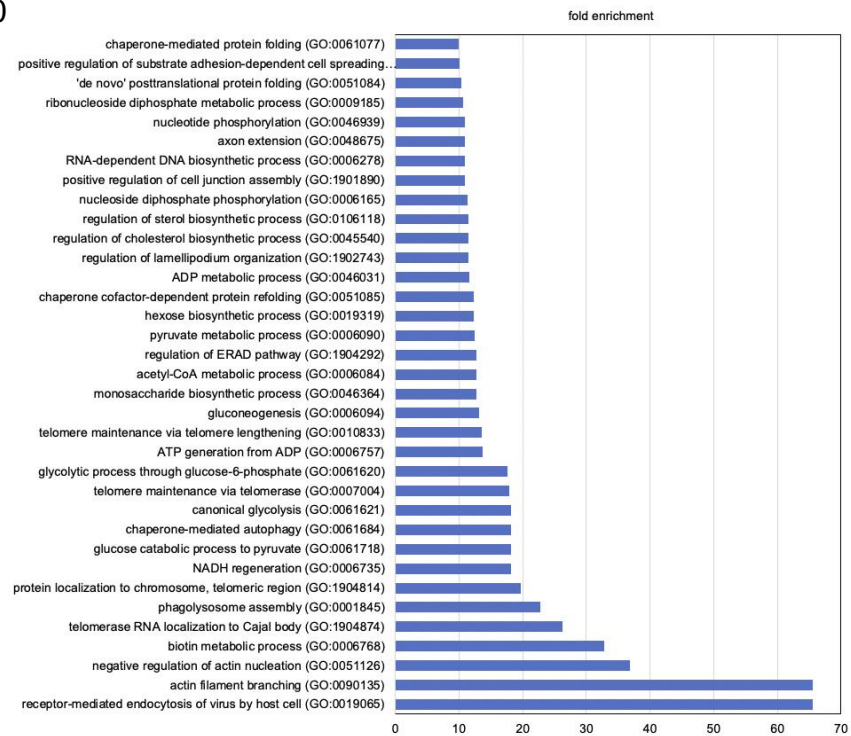

Supplement: S4 Fig — Fold enrichment values are shown for the top gene ontology categories among the HSC70 UBAIT targets (top panel) and the HSP70 UBAIT targets (bottom panel). FDR, p < 0.05 only. FDR, false discovery rate; HSC, heat shock cognate; HSP, heat shock protein; UBAIT, ubiquitin-activated interaction trap. (PDF) [file pbio.3000606.s004.pdf]

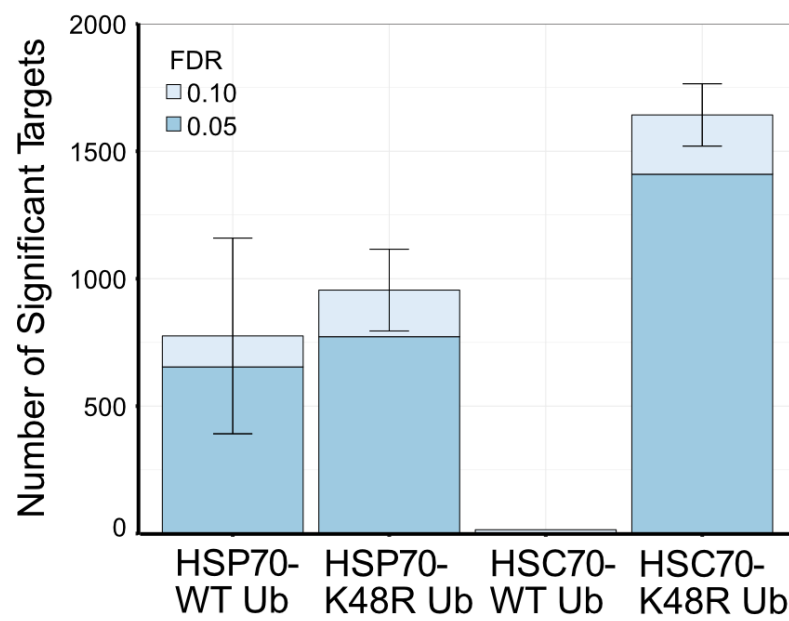

Supplement: S5 Fig — Error bars show standard deviation. See also S6 Data. HSC, heat shock cognate; HSP, heat shock protein; UBAIT, ubiquitin-activated interaction trap; WT, wild-type. (PDF) [file pbio.3000606.s005.pdf]

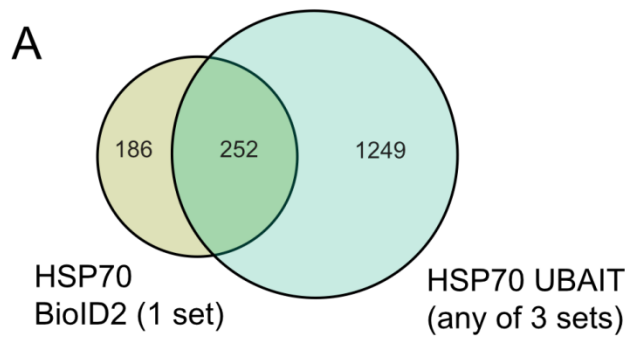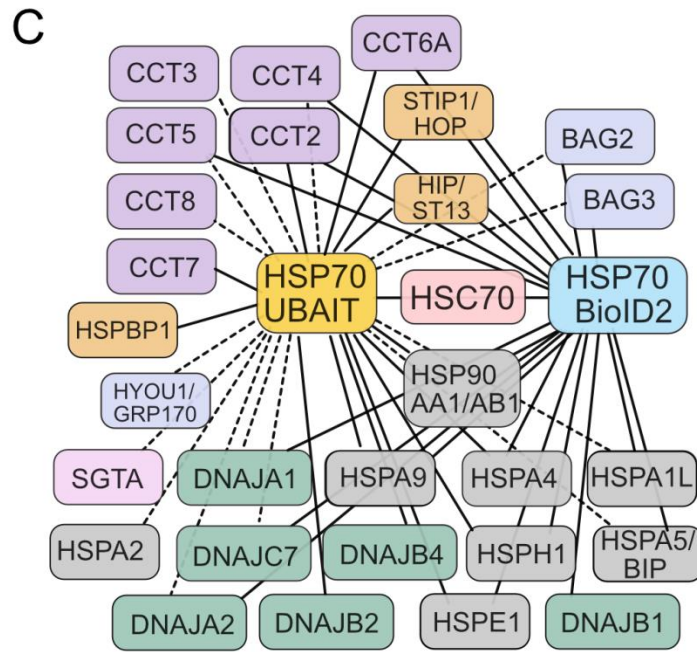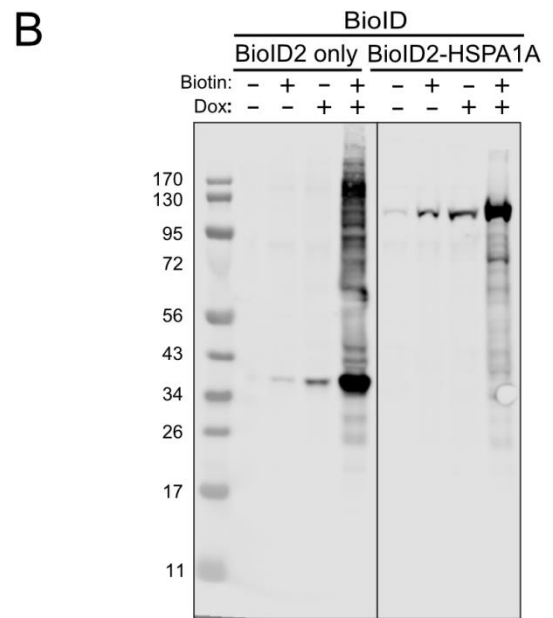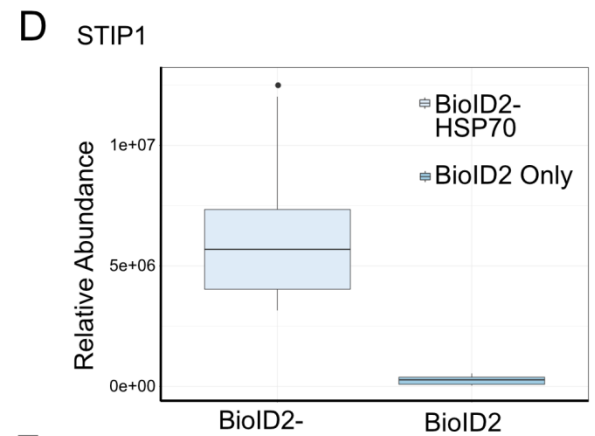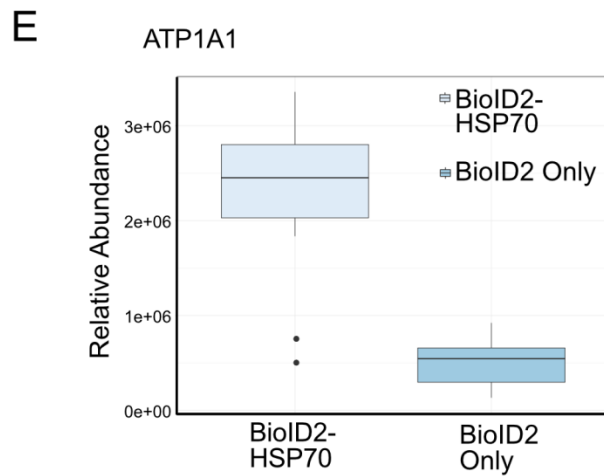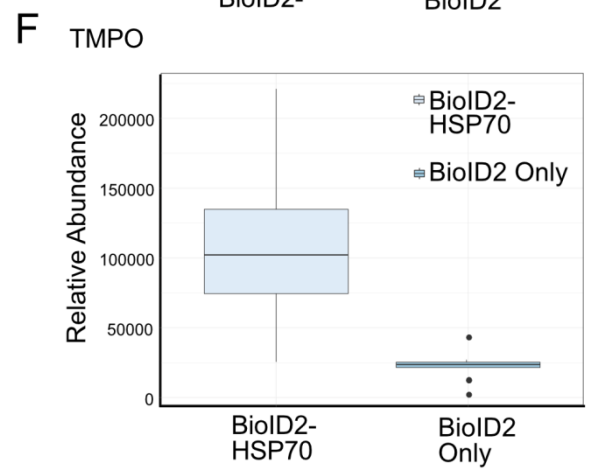

Supplement: S7 Fig — (A) Number of HSP70 binding partners identified by BioID2 (12 replicates [1 set]) compared to HSP70 UBAIT (significant at FDR 0.05 in at least one set). (B) Western blot of inducible BioID2 and BioID2-HSP70 fusion protein expression in U2OS cells, with biotin and doxycyline addition as indicated; visualized with streptavidin-AlexaFluor680 (Life Technologies). (C) Schematic diagram of targets identified through BioID2-HSP70 and UBAIT HSP70. Targets found in one or two sets (but not all three). UBAIT experiments are shown with a dashed line. (D, E, F) Levels of binding of specific targets to BioID2-HSP70 or BioID2 alone. All examples shown yield enrichment values that exceed the 95% confidence interval and are retained at FDR 0.05 using Benjamini-Hochberg (see Materials and methods for details). See also S6 Data. FDR, false discovery rate; HSP, heat shock protein; UBAIT, ubiquitin-activated interaction trap. (PDF) [file pbio.3000606.s007.pdf]
