# Supplementary material for: Proteome-wide identification of HSP70/HSC70 chaperone clients in human cells
Source: PLoS Biol. 2020 Jul 20;18(7):e3000606. doi: 10.1371/journal.pbio.3000606 (PMC7392334; doi:10.1371/journal.pbio.3000606)
Supplement: S6 Fig — (A) Venn diagrams of wild-type and V438F HSC70 UBAIT targets identified, each with N = 6, all with K48R ubiquitin fusions. (B) Western blot of HSC70 UBAITs expressed in human U2OS cells treated with doxycycline (Dox) (1 ug/mL) for 3 days or untreated, using streptavidin-AlexaFluor680 (Life Technologies). (C) Summary of average WALTZ [47] and TANGO [46] scores of significant targets, as well as polypeptide length of proteins enriched with UBAITs in cells expressing wild-type or V438F HSC70. Top: analysis including shared targets (239 WT versus 251 VF); bottom: analysis excluding shared targets (111 WT versus 123 VF). Welch’s one-tailed t test was used to compute p-values. HSC, heat shock cognate; UBAIT, ubiquitin-activated interaction trap; VF, V438F; WT, wild type. (PDF) [file pbio.3000606.s006.pdf]

A

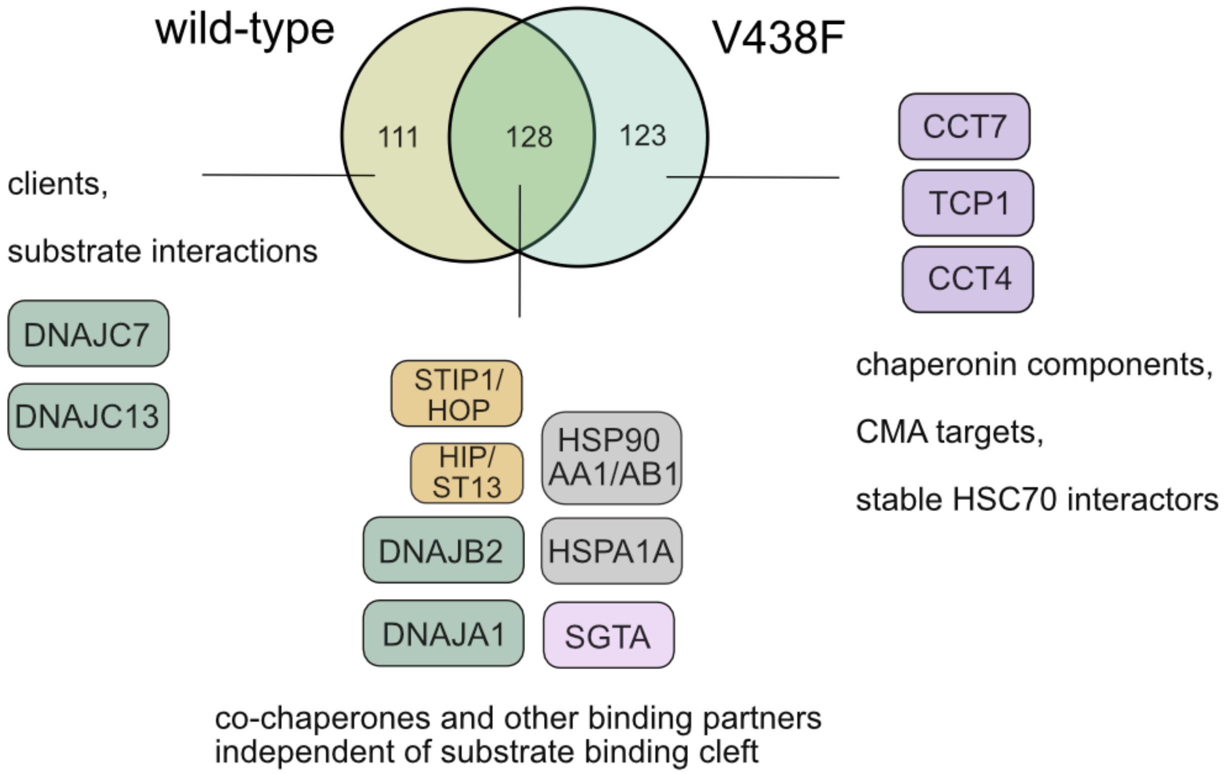

B

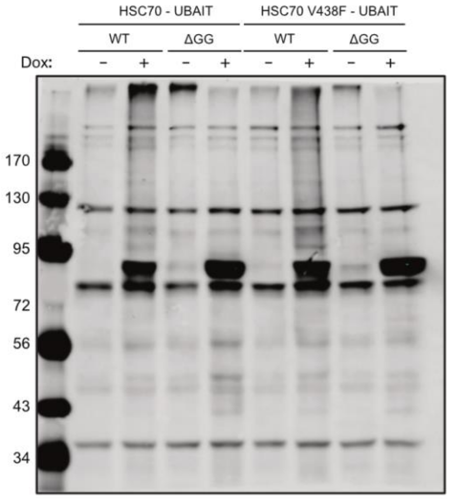

C

	HSC70		
	WT	V438F	P-Value
Waltz	1262.316	1088.312	0.06301
Tango	2546.139	1998.162	0.02512
AA Length	755.5443	612.3238	0.0179
	HSC70 (Excluding shared targets)		
	WT	V438F	P-Value
Waltz	1372.625	1003.146	0.01523
Tango	2852.515	1691.395	0.001672
AA Length	742.4455	444.547	1.44E-05
