# Supplementary material for: Proteome-wide identification of HSP70/HSC70 chaperone clients in human cells
Source: PLoS Biol. 2020 Jul 20;18(7):e3000606. doi: 10.1371/journal.pbio.3000606 (PMC7392334; doi:10.1371/journal.pbio.3000606)
Supplement: Attachment — Submitted filename: Reviewer Response 032820 final.pdf [file pbio.3000606.s014.pdf]

Thank you for the positive responses to our study. We have responded to all the points with the original reviewer text in blue below.

Rev. 1:
In this paper, Ryu et al profiled the clients of HSP70/HSC70 in human cells. They used a method named UBAIT, previously developed to capture interacting proteins of ubiquitin ligases, to identify HSP70/HSC70 interacting proteins. They show that HSP70/HSC70 have different client preference and a single misfolded protein SOD1 (A4V) induces changes in HSP70/HSC70 client association.

The major caveat in this study is that UBAIT was originally designed to study ubiquitin ligases (E3s) because E3s contain either a HECT or a RING domain that is necessary for ubiquitin transfer from E2 to substrates. However, HSC70 does not contain any characteristic E3 domains, thus when HSC70-Ub forms thioester with E2, it is unlikely to label HSC70 target directly. Instead, it will function with other E3s in cells, and potentially label other ubiquitin targets. It is possible that HSC70 and its targets are also labeled but the overall background will be significantly high. The cellular ubiquitination machineries will just take HSC70-Ub as a regular ubiquitin and incorporate HSC70-Ub into other ubiquitinated proteins in cells. The deltaGG control experiment cannot exclude this possibility since it is an inactive ubiquitin. The authors did profile all ubiquitinated proteins in cells using biotin-V5-ubiquitin only and claimed there are not much overlap between biotin-V5-ubiquitin and biotin-V5-HSC70-ubiquitin. However, only 52 unique proteins were identified with biotin-V5-ubiquitin. This is contradictory to many previous studies. Mass spectrometry can easily identify thousands of ubiquitinated proteins with tagged ubiquitin. Overall, the conclusions made in this paper seems all based on a method that is not validated to be useful for non-E3 proteins. The conclusions can alternatively be explained by the altered ubiquitination status of the "client" proteins. Here are other major concerns:

(1) The discovery and validation experiments are all based on UBAIT. First, the authors need to establish the proof of principle why this method can be used for non-E3s. Is there any covalent modification of known HSP70 client? How is the background? The current Biotin-V5-ubiquitin dataset is not convincing for the reason stated above. Secondly, the validation of the binding partners (Fig 3) should not use UBAIT since it is the method used to identify these potential binding partners. Alternative validation methods, such as co-IP, should be used for validation.

The reviewer is correct that the UBAIT approach was first designed and used for identifying substrates of HECT and RING domain ubiquitin ligases (O'Connor et al., 2015). However, a point made in that first report was that the ability of a HECT E3 UBAIT to trap bona fide interactors was independent of the ubiquitin ligase activity of the E3 (i.e., a HECT E3 with a mutant active-site expressed as a UBAIT trapped substrates as effectively as the wild-type HECT E3 UBAIT). This led O'Connor, et al. to propose that the UBAIT approach might not be limited to use with ubiquitin ligases but might also be useful for identifying interactors of a wide variety of proteins. A validation of this can be found in Swaim, et al., 2017, in which the UBAIT approach was used in an extracellular assay to identify the receptor for ISG15 (Swaim et al., 2017). In simplest terms, the UBAIT approach is a proximity ligation reaction in which the E2-activated ubiquitin moiety of the UBAIT is the donor (ligated) molecule. A comparison to another protein-protein interaction technique - the NEDDylator system – further makes this point. The key difference between UBAITs and NEDDylators is that the former is based on a Ub fusion to a bait protein and the latter on an E2 fusion to a bait protein. In both cases the trap is a Ub/NEDD8~E2 thioester, with the donor (ligated) molecule being Ub or NEDD8. Again, both of these approaches are independent of the bait being a ubiquitin ligase.

The reviewer is also correct that UBAITs can be potentially used as a source of ubiquitin in cells, although probably by only a subset of E2s and/or E3s (dependent on the particular UBAIT). This "side reaction" must be controlled for, which is why cells that expressed only biotin-V5-ubiquitin (without any bait protein) were analyzed in parallel. The concern of the reviewer is that only a small number of proteins were identified in the biotin-V5-ubiquitin analysis, and this clearly represent a tiny fraction of the total ubiquitinome. The primary reason for the small number of proteins identified with biotin-V5-ubiquitin is related to the strict thresholds that were used in statistical filtering, which removed proteins that did not have high levels of peptides relative to the deltaGG controls. Regardless, we are grateful to the reviewer for suggesting that a useful control in our study would be a substrate binding mutant of the HSC70 protein, and this control has been incorporated into the revised manuscript in Fig. S6. This, more than anything, confirms that the HSC70 UBAITs are identifying interacting proteins, rather than altering the ubiquitination status of HSC70 clients.

With respect to validation, we note that methods such as co-IPs are not sufficient to identify the large spectrum of chaperone binding partners; this is the primary reason for doing this study. We did do a complete set of BioID with HSP70 as an example of another method that also has the advantage of labeling many binding partners that accumulate over time (Fig. S7).

(2) Instead of deltaGG, a potential good control will be a HSP70 mutant that is defective in substrate binding.

The deltaGG mutant is used here to capture all of the non-covalent associations with the UBAIT, which gives us a very good quantitation of the background levels of binding to the bait protein and to the beads. These values are used to evaluate the statistical significance of targets bound to the wild-type ubiquitin UBAIT. An HSP70 mutant that is defective in substrate binding is a great idea though, and would potentially tell us what targets bind through the substrate binding cleft compared to other proteins that bind elsewhere. We addressed this by doing a UBAIT experiment with HSC70 V438F, a mutation previously shown to block substrate binding by HSP70 orthologs (Mayer et al., 2000). We did 6 replicates of each wild-type and V438F UBAIT isolation, along with 6 ΔGG isolations for each, for a total of 24 isolations plus lysates. This analysis yielded fewer binding partners due to the lower number of replicates, but we still recovered 239 targets for wild-type HSC70 and 251 for V438F. The V438F UBAIT showed a loss of 111 binding partners, the majority of which are likely to be clients. This experiment is now shown below in Response Figure 1 and in Fig. S6 in the main text.

In this experiment we found that 128 targets were shared between the wild-type and V438F isolations; these included the major co-chaperones: HSP90, HSPA1A/B, STIP1, ST13, and HSPA4, as well as DNAJB2 and SGTA. 111 binding partners of the wild-type enzyme were not recovered with the V438F version of the UBAIT. This group does include some co-chaperones, including the J domain proteins DNAJC7 and DNAJC13. Unlike canonical DnaJ family co-chaperones which bind to Hsp70's through their J domains and the nucleotide-binding domain of Hsp70 (Ahmad et al., 2011), DNAJC7(TPR2) associates with Hsp70 with contributions from the TPR domains in DNAJC7 and the substrate-binding domain of Hsp70 (Brychzy et al., 2003), which may explain why the binding of this J domain is sensitive to the V438F mutation. DNAJC13 (RME8) is also in this group of V438F-sensitive binding targets, perhaps due to its reported affinity for the ADP-bound (substrate-associated) form of HSC70 (Chang et al., 2004) in contrast to the ATP-bound HSC70 binding preference of most J proteins (Jiang et al., 2007; Mayer and Gierasch, 2019). Besides these co-chaperones, the 111 V438F-sensitive binding partners likely include client proteins.

A

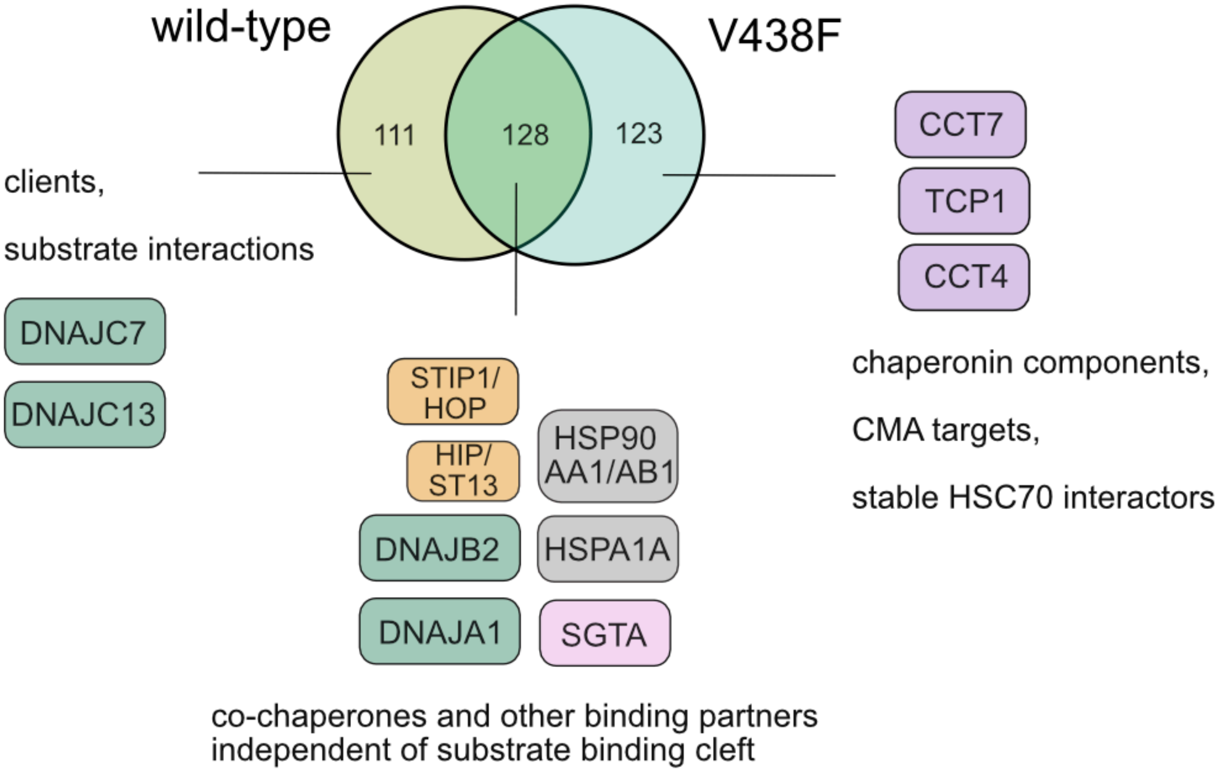

B

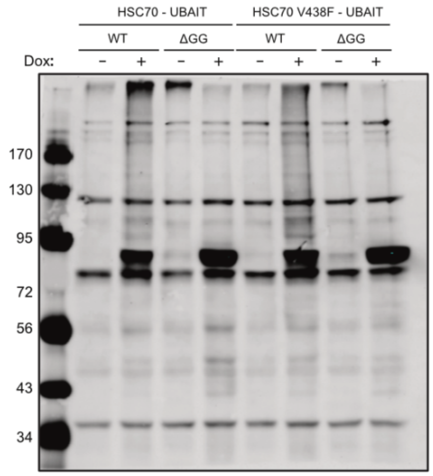

C

	HSC70		
	WT	V438F	P-Value
Waltz	1262.316	1088.312	0.06301
Tango	2546.139	1998.162	0.02512
AA Length	755.5443	612.3238	0.0179
	HSC70 (Excluding shared targets)		
	WT	V438F	P-Value
Waltz	1372.625	1003.146	0.01523
Tango	2852.515	1691.395	0.001672
AA Length	742.4455	444.547	1.44E-05

Response Figure 1. Significant targets identified from HSC70 UBAIT isolations containing either wild-type or V438F HSC70 ubiquitin. (A) Venn diagrams of wild-type and V438F HSC70 UBAIT targets identified, each with N=6, all with K48R ubiquitin fusions. (B) Western blot of HSC70 UBAITs expressed in human U2OS cells treated with doxycycline ("Dox")(1ug/ml) for 3 days or untreated, using Alexa Fluor 680 streptavidin. (C) Summary of average WALTZ [84] and TANGO [82] scores of significant targets as well as polypeptide length of proteins enriched with UBAITs in cells expressing wild-type or V438F HSC70. Top: analysis including shared targets (239 WT versus 251 VF; bottom: analysis excluding shared targets (111 WT versus 123 VF). Welch's one-tailed T test was used to compute p-values.

The group of prospective clients does not show any significant differences from the total proteome with respect to charge, but an analysis of the sequences using the TANGO and WALTZ algrorithms which predict propensity for amyloid and beta-sheet aggregation based on experimentally-derived parameters shows a significant difference between the V438F-sensitive targets of HSC70 in comparison to the targets bound by the V438F form of the UBAIT (Response Figure 1C). If the targets shared between these groups are removed, these differences are even more extreme, with the proposed client group shows TANGO, WALTZ, and length averages that are 40 to 70% higher than the group of targets that is bound only to the mutant UBAIT. Thus, one of the clear differences between the proposed client and non-client targets is propensity for aggregation.

The V438F mutant HSC70 UBAIT also preferentially bound to 123 targets that were not recovered at significant levels with the wild-type UBAIT (Response Figure 1). Examination of this list shows that several components of the chaperonin TRiC complex are represented here (TCP1, CCT7, and CCT4). This complex is known to bind to HSC70 (Knee et al., 2013; Lewis et al., 1992) and was shown to associate through the nucleotide-binding domain of the chaperone (Cuéllar et al., 2008), thus it is not surprising that the association is still observed with the mutant UBAIT. Why this is observed with the mutant and not the wild-type UBAIT is not clear, although it is possible that since a limited pool of UBAIT exists, the reduction in client binding through the substrate binding cleft allows for a larger subset of the UBAIT to associate with non-clients. Further analysis of this V438F-specific binding fraction shows that several proteins are known substrates for chaperone-mediated autophagy (CMA), a process by which HSC70 recognizes substrates through a conserved motif (KFERQ) and promotes its relocalization to the surface of lysosomes where it is internalized and degraded (Cuervo and Wong, 2014). The KFERQ binding motif does not bind to the HSC70 substrate binding cleft in the same way as canonical substrates (Taylor et al., 2018) and thus is likely not to be as affected by the V438F mutation. GAPDH, PLIN3, DPYSL2, and ANXA1 are all known to be regulated through CMA (Brekk et al., 2019; Kaushik and Cuervo, 2015; Liu et al., 2018; Pajares et al., 2018), so these and potentially other factors on this list of V438F-specific binding partners may be CMA targets. Other factors in this set are known to be stable binding partners of HSC70, likely through other interfaces (Guzhova et al., 2011; Hiyama et al., 2014; Hwang et al., 2018; Johansson et al., 2004; Kim et al., 2015; Liu et al., 2016; Matsui et al., 2019; Moghanibashi et al., 2013; Saitoh and Dasso, 1995; Tang et al., 2007; Xhabija and Vacratsis, 2015; Zhang et al., 2013). Overall, this analysis of the V438F interactome suggests that about half of the UBAIT targets are bound in a V438-sensitive manner, while half are not, with the latter group including co-chaperones as well as binding partners in other complexes.

(3) It is generally considered that chaperons assist protein folding, so it is not surprising that HSP70 partners are enriched for newly synthesized proteins. It is not obvious to the reader what proteins are regulated by HSP70 or HSC70 unless one checks the supplementary tables. It will be more informative to extend the study in Fig 2 to include more discussion about what are those proteins. Are those proteins enriched in particular biological pathway? Any functional relevance?

There are many biological pathways represented, most notably RNA-binding factors, metabolic enzymes, structural proteins, and chromosome-associated factors We have now summarized the most enriched pathways for both the HSC70 and HSP70 UBAIT experiments and their fold enrichment in Fig. S4.

(4) The paper also lacks the general information about what are the new HSP70 clients that are not previously identified.

The best identification of probable clients comes from the V438F experiment described above. The V438F-sensitive targets have a significantly higher level of predicted aggregation propensity, as

measured by the TANGO and WALTZ algorithms (Fernandez-Escamilla et al., 2004; Maurer-Stroh et al., 2010); there are no significant differences in charge or length of the polypeptides.

Minor concerns:
(1) Fig 1B, the molecular weight marker should be added.

This has been fixed.

(2) Fig 4E, is the y-axis the same as in Fig 3A? Why so different?

These values were originally normalized using a different method. They have now been corrected to use the same normalization.

(3) Duplications in the reference. Reference paper 25 is the same as 28.

Thanks - this has been fixed.

Rev. 2:
Ryo et al compare the substrate pool of human Hsp70 and Hsc70. The authors address a very important question, to which extent do Hsp70 homologues differ in substrate selectivity and hence in function. If such a case is convincingly made, it would be interesting for a broad readership. The authors identify substrates employing C-terminal ubiquitin fusions to subsequently create covalent substrate complexes that can be identified by mass spectrometry. The authors identify differences between Hsp70 and Hsc70. However, there are some serious concern that question the relevance of the finding and preclude publication in the present form.

Major concerns

1. The top hits identified by the authors contain highly positively charged proteins, many of which are nucleotide binding proteins. Others are proteins interacting with the ubiquitination machinery. As Hsp70s are strongly negatively charged, the identification of these substrates may be caused by coulomb interactions. This is even more likely as they work in overproduction conditions, favouring aberrant binding.

We have analyzed the targets for overall charge and length in comparison to the total proteome and to the unbound polypeptides in the lysate and there are no significant differences. The best estimate of a client group is the V438F-sensitive targets in the HSC70 UBAIT experiment now shown in Fig. S6. Here there are also no differences in charge but we do see a very obvious difference between the proposed clients and the non-client targets with respect to TANGO and WALTZ scores, which measure the amyloid and beta-sheet-based aggregation propensities of polypeptides using experimentally-based statistical algorithms (Fernandez-Escamilla et al., 2004; Maurer-Stroh et al., 2010). This is now discussed in detail in the main text and suggests that hydrophobic character and disorder propensity is a feature of HSC70 clients.

2. The authors need to include Hsp70 mutants allowing to comment on functional and specific interactions. This would be the classic mutations blocking ATP hydrolysis in the ATPase domain and the Val to Phe mutations blocking substrate binding via the substrate binding pocket.

This is an excellent idea; we performed a UBAIT with the V438F substrate binding pocket mutant and the results are described above in the response to Rev. 1 point 2 as well as in Fig. S6 and in

the main text.

3. The substrate data sets are poorly presented and analysed. The authors need to analyse to which protein classes their substrates belong to, and to which extent they identify substrates previously identified, and what would be common features (e.g. function, disorder, charge etc.).

The biological functions enriched in the UBAIT targets are now shown in Fig. S4. As discussed above, the best estimate for clients comes from V438F experiment, which is now presented in Fig. S6.

4. The C-terminal fusions preclude interaction with EEVD binding co-chaperones. It is unclear how and why the authors could identify some of the co-chaperones that bind to Hsp70 via this motif, e.g. Hop.

There are close interactions between Hsp70 and Hop but these proteins are also part of a ternary complex that forms between Hsp70, Hop, and HSP90 that includes multiple interfaces between Hop and the substrate-binding domain of Hsp70 (Alvira et al., 2014). This is consistent with findings that deletion of the EEVD motif in Hsp70 fails to disrupt association with Hop (Carrigan et al., 2004) and that motifs other than EEVD contribute to Hop complex formation (Brinker et al., 2002). Our recovery of Hop as a binding factor for both HSC70 and HSP70 despite the C-terminal fusion is thus not unexpected.

5. The authors claim the mutants are functional as Ub fusions complement in yeast. Such data need to be provided. They should be discussed keeping in mind that not all functions of Hsp70 chaperones under permissive conditions are essential functions

The yeast complementation data is now shown below in Response Figure 2 and in Fig. S2.

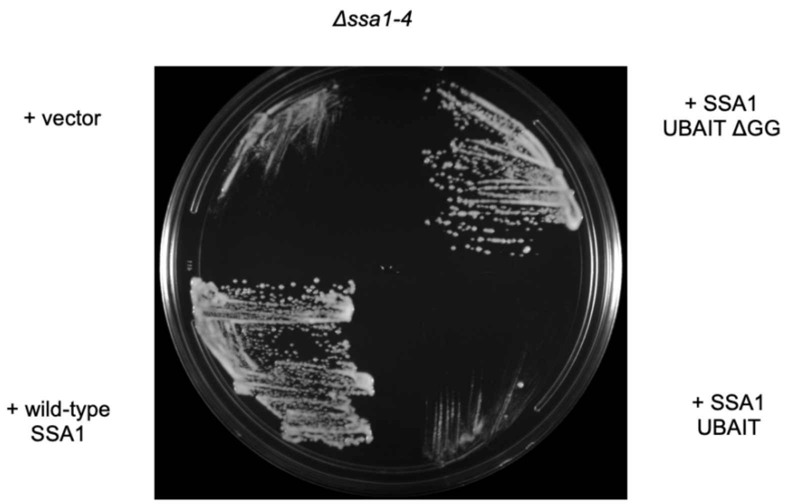

Response Figure 2. ΔGG UBAIT SSA1 complements *S. cerevisiae* deficient in HSP70 chaperones but wild-type UBAIT SSA1 does not.
An *S. cerevisiae* strain deficient in *SSA1, 2, 3*, and *4* was complemented by vector only, wild-type *SSA1*, *SSA1* UBAIT (C-term ubiquitin fusion), or *SSA1* UBAIT ΔGG (C-term ubiquitin fusion lacking GG at C-terminus), as indicated. Strains were streaked onto 5-FOA media, which selects for loss of the *URA3* (wt *SSA1*) plasmid maintaining viability of the ssa1-4 strain.

Rev. 3:
In this study, the authors employed a ubiquitin-mediated proximity ligation strategy (UBAIT) to covalently trap the binding partners of the human molecular chaperones HSC70 and HSP70. This system was originally utilized to map the interactors of ubiquitin ligases, but the K48R mutation makes it a versatile tool for other proteins of interest also. Like BioID and APEX, this approach can better capture direct, transient interactors compared to native affinity purification methods. Despite

the wealth of biochemical data on the Hsp70 family of chaperones, their endogenous substrates have not been systematically mapped. Ribosome profiling has been used to globally map co-translational Hsp70 clients in yeast, but no proteomic datasets were previously generated. Since all Hsp70 homologs that have been studied in vitro preferentially bind to hydrophobic peptide sequences, it is generally assumed that all paralogs in a given organism would have indistinguishable client repertoires.
However, by fusing human HSC70 and HSP70 to ubiquitin (K48R), expressing these constructs in HeLa cells, affinity purifying the chaperone-client conjugates and performing mass spectrometry, the authors found that HSC70 and HSP70 have a large set of non-overlapping clients. As expected based on the fact that in mature proteins hydrophobic binding sites are usually buried in protein cores, interaction interfaces and membranes, both HSC70 and HSP70 preferentially associate with nascent proteins and protein complex members lacking interaction partners. Finally, they show that that expression of an intrinsically misfolded protein (an ALS-associated SOD1 mutant) alters the landscape of HSC70 and HSP70 binding partners.

Comments
1) Overall the study is novel and provides a valuable resource that had been conspicuously absent in the literature.

2) The UBAIT strategy is a clever way to capture chaperone-client interactions.

3) The paper is well-written and the logic is easy to follow.

Thank you for the positive comments.

4) The ubiquitin fusion proteins are ectopically expressed on top of the wild type versions. The authors should assess what fraction of the total HSC70 and HSP70 is the UBAIT fusion.

To address this question we analyzed cells expressing the UBAIT fusions with an antibody directed against HSC70 (shown below in Response Figure 3 and in Fig. S1). This shows that the HSC70 UBAIT is expressed at levels substantially lower than the endogenous HSC70 level (comparing the ΔGG UBAIT to the endogenous band). The UBAIT fusion comprises approximately 10 to 15% of the total HSC70 in the cells. We do not have an antibody that is specific to HSP70 but we did compare the levels of the HSC70 and HSP70 UBAITs directly and find that these are comparable (Response Figure 3B, Fig. S1). This is important as we are directly comparing the HSC70 and HSP70 UBAITs in this study. Statements summarizing these results have been added to the main text.

A

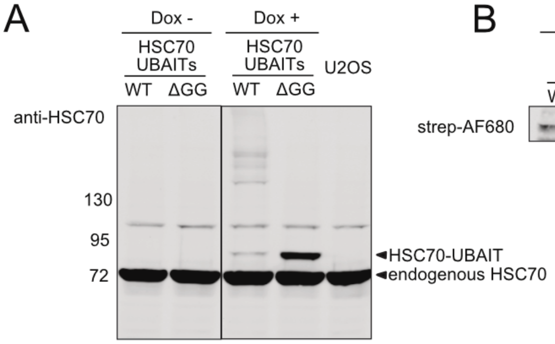

B

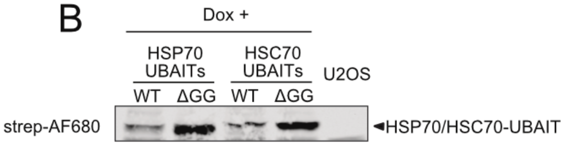

Response Figure 3. Levels of HSC70/HSP70 UBAITs are low in comparison to endogenous HSC70.
(A) Western blot of HSC70 UBAITs expressed in human U2OS cells treated with doxycycline ("Dox")(1ug/ml) for 3 days or untreated, using anti - HSC70 (Santa Cruz sc-7298). (B) Western blot of HSP70/HSC70 UBAITs expressed in human U2OS cells treated with doxycycline ("Dox")(1ug/ml) for 3 days or untreated, using Alexa Fluor 680 tagged streptavidin.

5) The authors should show the data demonstrating that HSC70-UBAIT is functional (complements the yeast deletion). The conserved "EEVD" sequence at the C-terminus is thought to be untaggable, so this should be commented on.

The yeast complementation data is shown above in response to Rev. 2, point #5 and in Fig. S2. The complementation was done with yeast *SSA1*, a constitutive chaperone that functions equivalently to HSC70 in humans.

6) HSP70 (HSPA1A) is not typically expressed under basal conditions, so the authors should caveat that the HSP70 results are not physiological.

A comment about this has been added to the main text.

7) The authors should comment on the identification of ER resident proteins (e.g., DNAJC proteins). Is this occurring post-lysis, or is the protein getting into the ER?

The cells are lysed in 8 M urea so we are quite sure that it does not happen post-lysis. We envision that many targets are bound during or shortly after translation, based on the SILAC data presented in Fig. 2.

8) It would be reassuring to show by IF that the UBAIT tagging does not alter the subcellular localization of HSC70/HSP70.

To address this question we imaged HSC70 and HSP70 by immunofluorescence in normal U2OS cells and U2OS cells expressing the HSC70 and HSP70 UBAIT genes, as shown below in Response Figure 4 and in Fig. S1. (The antibody for HSC70 is specific for HSC70 but the HSP70 antibody recognizes HSP70 and HSC70.) There are no significant differences between the chaperone distributions that are discernible by this method.

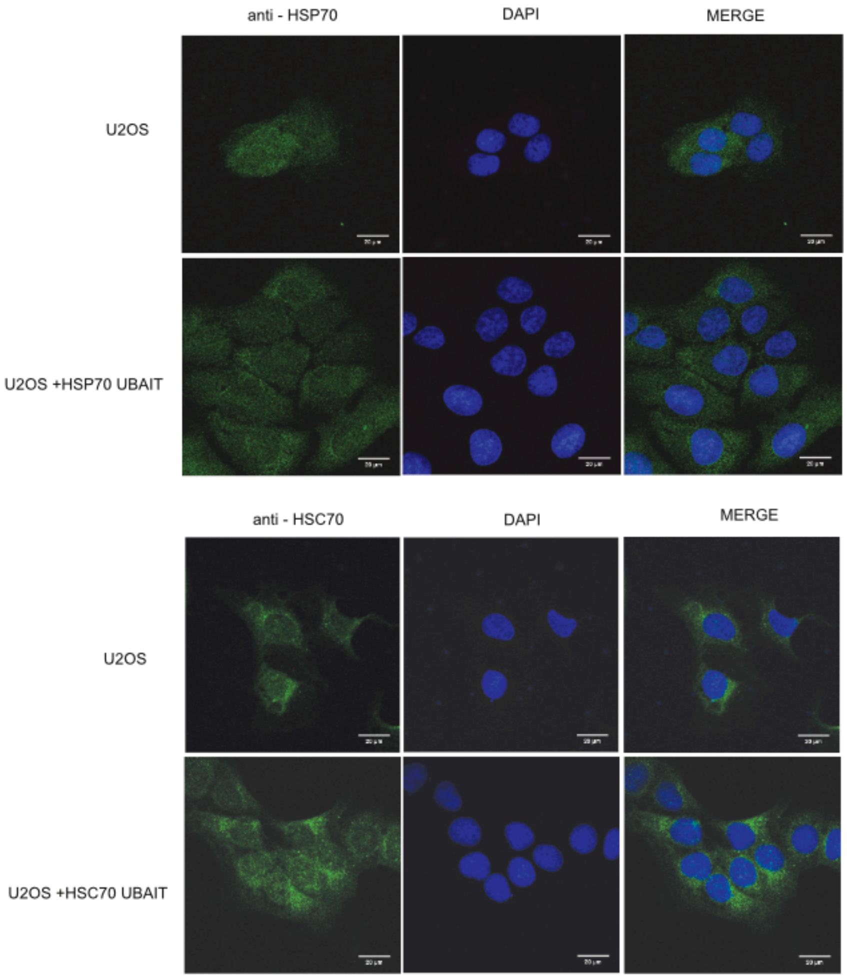

Response Figure 4. Visualization of HSC70 and HSP70 UBAIT proteins in human cells by immunofluorescence. U2OS cells and U2OS cells expressing the ubiquitin-tagged UBAIT constructs (with doxycycline) were analyzed by immunofluorescence using antibodies directed against HSP70 (Enzo ADI-SPA-810) or HSC70 (Santa Cruz sc-7298) and imaged by confocal microscopy with DAPI as the counterstain, as indicated.

9) The word "comprehensive" in the title is too strong and impossible to verify. Moreover, multiple cell lines were not examined. "Proteome-wide identification of HSP70/HSC70 chaperone clients in a human cell line" would be more accurate.

The title has been changed in response to this suggestion.

10) The comparison of the BioID vs UBAIT strategies is imperfect since the author utilized different termini of HSP70 (C terminal for UBAIT and N terminal for BioID2) for tagging. Since the BioID tag is on the N-terminus connected to the ATPase domain, it is more likely to get all the cochaperones (evidenced by DNAJB1), but farther from the substrate binding domain at the C-terminus. This should be caveated.

A comment about this has been added to the main text.

11) Also, a detailed method for the BioID approach is lacking.

The BioID isolation method is identical to the UBAIT isolation method since all of the targets are biotinylated. This has been clarified in the methods section, and additional information has been added to the statistical methods section related to BioID.

Minor Comments
1) Labelling:
a) label is missing in the bar graph of Figure 3C.
b) In all Figures for consistency label use either dGG or ∆GG.
c) Figure 4B, be consistent with HSP70 UBAIT or HSP70 Ubait

Thanks; these have been fixed.

References cited:

Ahmad, A., Bhattacharya, A., McDonald, R.A., Cordes, M., Ellington, B., Bertelsen, E.B., and Zuiderweg, E.R.P. (2011). Heat shock protein 70 kDa chaperone/DnaJ cochaperone complex employs an unusual dynamic interface. Proc. Natl. Acad. Sci. *108*, 18966–18971.

Alvira, S., Cuéllar, J., Röhl, A., Yamamoto, S., Itoh, H., Alfonso, C., Rivas, G., Buchner, J., and Valpuesta, J.M. (2014). Structural characterization of the substrate transfer mechanism in Hsp70/Hsp90 folding machinery mediated by Hop. Nat. Commun. *5*, 5484.

Brekk, O.R., Makridakis, M., Mavroeidi, P., Vlahou, A., Xilouri, M., and Stefanis, L. (2019). Impairment of chaperone-mediated autophagy affects neuronal homeostasis through altered expression of DJ-1 and CRMP-2 proteins. Mol. Cell. Neurosci. *95*, 1–12.

Brinker, A., Scheufler, C., Von Der Mulbe, F., Fleckenstein, B., Herrmann, C., Jung, G., Moarefi, I., and Hartl, F.U. (2002). Ligand discrimination by TPR domains. Relevance and selectivity of EEVD-recognition in Hsp70 x Hop x Hsp90 complexes. J. Biol. Chem. *277*, 19265–19275.

Brychzy, A., Rein, T., Winklhofer, K.F., Hartl, F.U., Young, J.C., and Obermann, W.M.J. (2003). Cofactor Tpr2 combines two TPR domains and a J domain to regulate the Hsp70/Hsp90 chaperone system. EMBO J. *22*, 3613–3623.

Carrigan, P.E., Nelson, G.M., Roberts, P.J., Stoffer, J., Riggs, D.L., and Smith, D.F. (2004). Multiple domains of the co-chaperone Hop are important for Hsp70 binding. J. Biol. Chem. *279*, 16185–16193.

Chang, H.C., Hull, M., and Mellman, I. (2004). The J-domain protein Rme-8 interacts with Hsc70 to control clathrin-dependent endocytosis in Drosophila. J. Cell Biol. *164*, 1055–1064.

Cuéllar, J., Martín-Benito, J., Scheres, S.H.W., Sousa, R., Moro, F., López-Viñas, E., Gómez-Puertas, P., Muga, A., Carrascosa, J.L., and Valpuesta, J.M. (2008). The structure of CCT-Hsc70 NBD suggests a mechanism for Hsp70 delivery of substrates to the chaperonin. Nat. Struct. Mol. Biol. *15*, 858–864.

Cuervo, A.M., and Wong, E. (2014). Chaperone-mediated autophagy: roles in disease and aging. Cell Res. *24*, 92–104.

Fernandez-Escamilla, A.-M., Rousseau, F., Schymkowitz, J., and Serrano, L. (2004). Prediction of sequence-dependent and mutational effects on the aggregation of peptides and proteins. Nat. Biotechnol. *22*, 1302–1306.

Guzhova, I.V., Lazarev, V.F., Kaznacheeva, A.V., Ippolitova, M.V., Muronetz, V.I., Kinev, A.V., and Margulis, B.A. (2011). Novel mechanism of Hsp70 chaperone-mediated prevention of polyglutamine aggregates in a cellular model of huntington disease. Hum. Mol. Genet. *20*, 3953–3963.

Hiyama, G., Matsuzaki, M., Mizushima, S., Dohra, H., Ikegami, K., Yoshimura, T., Shiba, K., Inaba, K., and Sasanami, T. (2014). Sperm activation by heat shock protein 70 supports the migration of sperm released from sperm storage tubules in Japanese quail (Coturnix japonica). Reprod. Camb. Engl. *147*, 167–178.

Hwang, I., Cho, S.-W., and Ahn, J.-Y. (2018). Chaperone-E3 Ligase Complex HSP70-CHIP Mediates Ubiquitination of Ribosomal Protein S3. Int. J. Mol. Sci. *19*.

Jiang, J., Maes, E.G., Taylor, A.B., Wang, L., Hinck, A.P., Lafer, E.M., and Sousa, R. (2007). Structural basis of J cochaperone binding and regulation of Hsp70. Mol. Cell *28*, 422–433.

Johansson, T., Grenklo, S., and Karlsson, R. (2004). Detection of binding partners to the profilin:actin complex by far Western and mass spectrometry analyses. Anal. Biochem. *335*, 228–234.

Kaushik, S., and Cuervo, A.M. (2015). Degradation of lipid droplet-associated proteins by chaperone-mediated autophagy facilitates lipolysis. Nat. Cell Biol. *17*, 759–770.

Kim, J.Y., Yenari, M.A., and Lee, J.E. (2015). Regulation of inflammatory transcription factors by heat shock protein 70 in primary cultured astrocytes exposed to oxygen-glucose deprivation. Neuroscience *286*, 272–280.

Knee, K.M., Sergeeva, O.A., and King, J.A. (2013). Human TRiC complex purified from HeLa cells contains all eight CCT subunits and is active in vitro. Cell Stress Chaperones *18*, 137–144.

Lewis, V.A., Hynes, G.M., Zheng, D., Saibil, H., and Willison, K. (1992). T-complex polypeptide-1 is a subunit of a heteromeric particle in the eukaryotic cytosol. Nature *358*, 249–252.

Liu, L., An, D., Xu, J., Shao, B., Li, X., and Shi, J. (2018). Ac2-26 Induces IKKβ Degradation Through Chaperone-Mediated Autophagy Via HSPB1 in NCM-Treated Microglia. Front. Mol. Neurosci. *11*, 76.

Liu, Y., Jiang, S., Yang, P.-Y., Zhang, Y.-F., Li, T.-J., and Rui, Y.-C. (2016). EF1A1/HSC70 Cooperatively Suppress Brain Endothelial Cell Apoptosis via Regulating JNK Activity. CNS Neurosci. Ther. *22*, 836–844.

Matsui, H., Hazama, S., Tamada, K., Udaka, K., Irie, A., Nishimura, Y., Miyakawa, T., Doi, S., Nakajima, M., Kanekiyo, S., et al. (2019). Identification of a Promiscuous Epitope Peptide Derived From HSP70. J. Immunother. Hagerstown Md 1997 *42*, 244–250.

Maurer-Stroh, S., Debulpaep, M., Kuemmerer, N., de la Paz, M.L., Martins, I.C., Reumers, J., Morris, K.L., Copland, A., Serpell, L., Serrano, L., et al. (2010). Exploring the sequence determinants of amyloid structure using position-specific scoring matrices. Nat. Methods *7*, 237–242.

Mayer, M.P., and Gierasch, L.M. (2019). Recent advances in the structural and mechanistic aspects of Hsp70 molecular chaperones. J. Biol. Chem. *294*, 2085–2097.

Mayer, M.P., Schröder, H., Rüdiger, S., Paal, K., Laufen, T., and Bukau, B. (2000). Multistep mechanism of substrate binding determines chaperone activity of Hsp70. Nat. Struct. Biol. *7*, 586–593.

Moghanibashi, M., Rastgar Jazii, F., Soheili, Z.-S., Zare, M., Karkhane, A., Parivar, K., and Mohamadynejad, P. (2013). Esophageal cancer alters the expression of nuclear pore complex binding protein Hsc70 and eIF5A-1. Funct. Integr. Genomics *13*, 253–260.

O'Connor, H.F., Lyon, N., Leung, J.W., Agarwal, P., Swaim, C.D., Miller, K.M., and Huibregtse, J.M. (2015). Ubiquitin-Activated Interaction Traps (UBAITs) identify E3 ligase binding partners. EMBO Rep. *16*, 1699–1712.

Pajares, M., Rojo, A.I., Arias, E., Díaz-Carretero, A., Cuervo, A.M., and Cuadrado, A. (2018). Transcription factor NFE2L2/NRF2 modulates chaperone-mediated autophagy through the regulation of LAMP2A. Autophagy *14*, 1310–1322.

Saitoh, H., and Dasso, M. (1995). The RCC1 protein interacts with Ran, RanBP1, hsc70, and a 340-kDa protein in Xenopus extracts. J. Biol. Chem. *270*, 10658–10663.

Swaim, C.D., Scott, A.F., Canadeo, L.A., and Huibregtse, J.M. (2017). Extracellular ISG15 Signals Cytokine Secretion through the LFA-1 Integrin Receptor. Mol. Cell *68*, 581-590.e5.

Tang, D., Kang, R., Xiao, W., Jiang, L., Liu, M., Shi, Y., Wang, K., Wang, H., and Xiao, X. (2007). Nuclear heat shock protein 72 as a negative regulator of oxidative stress (hydrogen peroxide)-induced HMGB1 cytoplasmic translocation and release. J. Immunol. Baltim. Md 1950 *178*, 7376–7384.

Taylor, I.R., Ahmad, A., Wu, T., Nordhues, B.A., Bhullar, A., Gestwicki, J.E., and Zuiderweg, E.R.P. (2018). The disorderly conduct of Hsc70 and its interaction with the Alzheimer's-related Tau protein. J. Biol. Chem. *293*, 10796–10809.

Xhabija, B., and Vacratsis, P.O. (2015). Receptor-mediated Endocytosis 8 Utilizes an N-terminal Phosphoinositide-binding Motif to Regulate Endosomal Clathrin Dynamics. J. Biol. Chem. *290*, 21676–21689.

Zhang, L., Liu, Q., Yuan, X., Wang, T., Luo, S., Lei, H., and Xia, Y. (2013). Requirement of heat shock protein 70 for inducible nitric oxide synthase induction. Cell. Signal. *25*, 1310–1317.
