# Supplementary material for: Proteome-wide identification of HSP70/HSC70 chaperone clients in human cells
Source: PLoS Biol. 2020 Jul 20;18(7):e3000606. doi: 10.1371/journal.pbio.3000606 (PMC7392334; doi:10.1371/journal.pbio.3000606)
Supplement: Attachment — Submitted filename: Reviewer Response 060920.docx [file pbio.3000606.s015.docx]

**Reviewer Response**

Rev. 1 and 3: Thank you for the positive response to our revised manuscript.

Rev. 2:

1. The V438F substrate binding pocket mutation does not lessen the ability of the UBAIT fusion to conjugate to associated proteins, it just lowers the affinity of the binding pocket for some substrates. So with a V438F mutant form of the UBAIT, one would expect more associations with binding partners that are not bound through the substrate-binding pocket, which is what we observed.
2. The data files contain all UNIPROT IDs and gene names for the targets isolated in all experiments; thus all the data is available for readers to assess properties of these targets.
3. It is not clear to us what would be gained by testing an ATPase-deficient mutant. We thought that the V438F experiment was an excellent suggestion and thus focused our additional time, effort, and funds on this rather than the ATPase-deficient mutant.
